# Stabilization of yield in plant genotype mixtures through compensation rather than complementation

**DOI:** 10.1093/aob/mct209

**Published:** 2013-09-18

**Authors:** Henry E. Creissen, Tove H. Jorgensen, James K. M. Brown

**Affiliations:** 1 Crop Genetics Department, John Innes Centre, Norwich Research Park, Norwich, Norfolk NR4 7UH, UK; 2 School of Biological Sciences, University of East Anglia, Norwich Research Park, Norwich, Norfolk NR4 7TJ, UK; 3 Department of Bioscience, Aarhus University, Aarhus, Denmark

**Keywords:** *Arabidopsis thaliana*, compensation, experimental ecology, genotype mixtures, model-to-crop translational research, plant competition, resistance to environmental stress, variety mixtures, yield stability

## Abstract

**Background and Aims:**

Plant genotypic mixtures have the potential to increase yield stability in variable, often unpredictable environments, yet knowledge of the specific mechanisms underlying enhanced yield stability remains limited. Field studies are constrained by environmental conditions which cannot be fully controlled and thus reproduced. A suitable model system would allow reproducible experiments on processes operating within crop genetic mixtures.

**Methods:**

Phenotypically dissimilar genotypes of *Arabidopsis thaliana* were grown in monocultures and mixtures under high levels of competition for abiotic resources. Seed production, flowering time and rosette size were recorded.

**Key Results:**

Mixtures achieved high yield stability across environments through compensatory interactions. Compensation was greatest when plants were under high levels of heat and nutrient stress. Competitive ability and mixture performance were predictable from above-ground phenotypic traits even though below-ground competition appeared to be more intense.

**Conclusions:**

This study indicates that the mixing ability of plant genotypes can be predicted from their phenotypes expressed in a range of relevant environments, and implies that a phenotypic screen of genotypes could improve the selection of suitable components of genotypic mixtures in agriculture intended to be resilient to environmental stress.

## INTRODUCTION

Empirical studies have shown that higher levels of plant species diversity can result in greater above-ground productivity (Hector *et al.*, 1999; van Ruijven and Berendse, 2005; Roscher *et al.*, 2011) and ecosystem stability (Tilman *et al.*, 2006). Previous studies on the relationships between plant diversity, stability and productivity of ecosystems have focused on diversity at the species level (Tilman, 2001), yet these relationships are also observed at the functional group and genotype level (Hector *et al.*, 1999; Hughes and Stachowicz, 2011). The potential of plant diversity to increase or stabilize productivity is of great interest in crop systems (Zhu *et al.*, 2000; Li *et al.*, 2009). However, there is limited understanding of the actual mechanisms leading to correlations between plant diversity, productivity and stability which currently restricts the use of biologically diverse cropping systems in agriculture.

Ecological stability is commonly described using two main terms, resistance and resilience. Resistance refers to the ability of the system to resist change in response to perturbation, whereas resilience refers to the ability of the system to recover by returning to its pre-perturbation state (for reviews, see Tilman, 1996; Hooper *et al.*, 2005). Resistance is the more relevant trait in annual plants, particularly when environmental stress occurs near or after the time of flowering. Note that resistance in the ecological sense used here, operating at the population or community level, is not the same as resistance of individual plants to stress or disease. Proposed mechanisms by which stability is achieved by ecological resistance in diverse communities or populations include compensation, complementation and facilitation. Compensation occurs when a species displays resistance to perturbation and is able to compensate for more susceptible species. It requires variation between species or genotypes in response to stress and competition, allowing the stronger species or genotypes to compensate for weaker ones via competitive release (Tilman, 1996). Such interactions increase stability in productivity at the community level but increase variability at the population and species level (Tilman *et al.*, 1996; Bai *et al.*, 2004). Similar compensatory mechanisms may occur between genotypes in a diverse population of a single species (McLaren and Turkington, 2011). Complementation, on the other hand, results from increased resource use efficiency in mixed communities or populations because individual plants often experience less niche overlap than in monoculture, which can lead to over-yielding in species mixtures (Hector *et al.*, 2002; Silvertown, 2004). Finally, facilitation results from positive interactions between species or genotypes, which may increase productivity and stability by altering features of the local environment to the benefit of neighbouring plants, such as the accumulation of nutrients, provision of shade and protection from herbivores (Callaway, 2002). Facilitation is indicated if plants perform significantly worse when a neighbour is removed and is common in stressful environments (Callaway, 2002; Kikvidze *et al.*, 2006).

Crop breeding programmes produce cultivars with increased yield potential which must be coupled with improved farming practices to achieve those yields (Calderini and Slafer, 1998). In most situations, a single cultivar that is completely or almost completely genetically uniform is grown throughout a field (Trewavas, 2001). Monocultures rely heavily on chemical inputs such as fungicides, pesticides and herbicides to maintain the specific environment required for successful cropping. However, selection for performance under high input conditions and low environmental variation can lead to a reduction in yield stability across environments (Calderini and Slafer, 1999). The use of agro-chemicals may be heavily restricted in the future, forcing farmers to consider using alternative cropping systems that are adaptable to multiple environments (Hillocks, 2012). If plant diversity within fields of agricultural crops contributes to achieve stable, high levels of production, it will promote food security, which is threatened by a ‘perfect storm’ of multiple interacting environmental and natural resource challenges (Beddington, 2009).

Considering the current threat of global warming and the unpredictable ecological responses to climate change (Lavergne *et al.*, 2010), the importance of increasing the adaptive power of crops is of great concern (Lobell, 2008). Varietal mixtures, where several cultivars are grown together, are only used to a limited extent in modern, intensive farming owing to perceived disadvantages regarding heterogeneity of the end-product and variable agronomy (Newton *et al.*, 2009). Mixtures have the potential to increase yield stability and control pests and diseases whilst being less reliant on chemical inputs which generate a high demand for energy in their production and application (Wolfe, 1985; Altieri, 1999; Zhu *et al.*, 2000).

Presently, evidence for the advantages and disadvantages of growing varietal mixtures comes from studies that are typically large in scale because of the high variances associated with the uncontrolled environment and genotype by environment interactions (Madden *et al.*, 2007). A suitable model system in which environmental conditions are more readily controlled would require fewer plants, making it feasible to manipulate and test the effects of specific interactions and to obtain insights into the mechanisms at work in crop mixture systems. Greater understanding of the plant–plant interactions within varietal mixtures and the interaction of the crops with the environment has the potential to inform rational choices of component varieties in mixtures.

*Arabidopsis thaliana* (Brassicaceae) is a small annual weed that has been successfully used as a model for understanding plant biology (Mitchell-Olds, 2001; Jorgensen, 2012; Meldau *et al.*, 2012). Arabidopsis, like most weedy species, is an *r*-strategist producing thousands of small seeds with little investment of resources per seed (MacArthur and Wilson, 1967). It occurs naturally in highly disturbed environments with little competition, but it can readily be used in competition studies because genotypes can differ greatly in biomass, seed production, resource requirements and competitive ability (Cahill *et al.*, 2005; Masclaux *et al.*, 2010). Phenotypic variation for traits relating to competitive ability observed within a genotype can be largely attributed to environmental variation (Clauss and Aarssen, 1994), and several studies have found significant interactions between genotypes and environments (Pigluici *et al.*, 1995*a*, *b*). The small size of arabidopsis plants and short generation times under glasshouse conditions provide a model system in which the high levels of replication required for competition experiments across environments can be reliably achieved. These attributes may make arabidopsis a powerful tool for controlled ecological studies on competition between plants.

Here we examine arabidopsis as a model system to study the effects of genotypic diversity on yield under glasshouse conditions. The roles of compensation and complementation in stabilizing productivity in genotypic mixtures of arabidopsis were determined for plants subjected to the types of abiotic stresses that may challenge present and future agricultural systems. We tested the hypotheses that: (1) genotypic mixtures have greater yield stability than monocultures, particularly when under environmental stress; (2) the yield of individual genotypes is more variable within mixtures than monocultures but compensation by stronger competitors within the mixtures begets an increase in yield stability for the mixture as a whole; and (3) competitive ability can be predicted from plant phenotype.

## MATERIALS AND METHODS

### Four-way mixture experiments

Four genotypes of *Arabidopsis thaliana* were selected for study (Ler-1, Col-0, Gy-0 and Ga-0) based largely on phenotypic variation for rosette size and seed production. Genotypes were selected to vary in flowering time by a few days at most so they would compete for resources at a similar time. Four-way mixture experiments were conducted to investigate the effects of all the genotypes competing with each other. The experiment was conducted in large trays (680 × 440 × 50 mm) in which inter-plant distance was 30 mm for horizontally and laterally nearest neighbours, and 40 mm between diagonally opposite neighbours, which generated intense competition between plants. In the absence of competitors under optimal growing conditions, genotypes ranged in rosette diameter from 3 to 11 cm. Plants were cultivated as both monocultures and four-way mixtures in which competition between genotypes was intensified by maximizing the distance between plants of the same genotype (Supplementary Data Fig. S1). Seeds were sown in Levington F2 soil and were incubated at 4 °C for 4 d to break dormancy before being moved to the glasshouse at 21–23 °C on a 16 h light/8 h dark cycle supplemented with 120 μmol m^−2^ s^−1^ fluorescent lighting for germination. After 7 d, seedlings were transplanted into the experimental layout. Plants were grown under high or low nutrient conditions from the seedling stage until senescence. The high nutrient treatment consisted of eight parts peat-based compost to one part grit. Low nutrient conditions were created by diluting the high nutrient soil mixture with medium grade (2–5 mm) vermiculite (1:2 v/v).

Each experimental replicate consisted of two replicates of each of the four monocultures under both nutrient conditions, and six replicates of four-way mixtures per nutrient condition, resulting in a total of 28 trays per replicate. For each monoculture tray, ten focal plants were randomly selected for phenotypic trait analysis, whereas for each mixture tray, ten focal plants of each genotype were sampled.

Two independent experiments were performed during autumn (beginning in October 2010) and winter (beginning in January 2011). Both experiments (autumn and winter) had additional lighting for the duration of the experiment. Temperatures were fairly constant (mean temperature 19/20 °C, daily maximum 26/27 °C, standard deviation of mean temperature 1·3/1·8 °C) during these replications. Another experiment ran during summer (beginning in June 2010) using the same design as the other two but it was exposed to additional heat stress, not replicated in other seasons. Temperature and light levels were substantially higher than in the other two experiments (mean temperature 21 °C, daily maximum 31 °C, standard deviation of mean temperature 2·6 °C). No additional lighting was provided during the summer experiment.

Several measurements were taken for each focal plant to assess plant fitness, including days to first flower (phase 6, Boyes *et al.*, 2001), height of longest inflorescence at the onset of silique maturation and total seed mass. Plants were bagged with individual clear, micro-perforated bags when the first siliques began to ripen to ensure all seeds were collected. Relative yield (yield in mixture/yield in monoculture) (de Wit, 1960) was calculated for each genotype to assess mixture performance.

### Pair-wise interaction experiments

To test the hypothesis that competitive ability can be predicted from above-ground phenotypic traits, pair-wise interaction experiments were conducted to investigate the effect of a single competing genotype on the fitness of the focal genotype. Plants were treated as focal or competing, but not both, because it was not possible to bag adjacent plants for seed collection. The four genotypes (Ler-1, Col-0, Gy-0 and Ga-0) and a different set of four genotypes (Wei-0, Van-0, Ms-0 and Ema-1) were assigned to a competitive group based on phenotypic traits relating to their predicted competitive ability such as seed production, rosette size and flowering time when grown as a single plant (Table 1). Genotypes received a ranking for each trait. These rankings were weighted to calculate predicted competitive ability; seed mass was given a weighting of 4, rosette size a weight of 2 and flowering time a weight of 1. Group 1 had the lowest predicted competitive ability due to its low yield, small rosette and early flowering, whereas group 4 was predicted to be the most competitive.

**Table 1.** Mean trait values (± s.d.) for eight arabidopsis genotypes grown under high nutrient conditions in the absence of competitors (*n* = 5 plants per genotype)

Set	Competitive group	Genotype	Days to flower	Rosette diameter at 4 weeks (mm)	Seed mass (g)
1	1	Ler-1	25·0 ± 0·0	28·5 ± 3·1	0·019 ± 0·008
1	2	Col-0	26·6 ± 1·7	57·4 ± 5·6	0·124 ± 0·013
1	3	Gy-0	31·0 ± 2·6	98·8 ± 21·4	0·177 ± 0·074
1	4	Ga-0	28·6 ± 2·7	84·4 ± 12·9	0·345 ± 0·079
2	1	Van-0	29·0 ± 0·0	34·6 ± 4·2	0·090 ± 0·013
2	2	Wei-0	25·8 ± 1·1	60·0 ± 11·9	0·098 ± 0·041
2	3	Ms-0	25·7 ± 1·2	50·4 ± 14·9	0·127 ± 0·074
2	4	Ema-1	34·0 ± 4·0	111·3 ± 10·7	0·516 ± 0·093

Growing conditions were the same as in the high nutrient treatment of the four-way experiment except that plants were grown in small pots (70 × 70 × 70 mm), each of which contained four plants. Plants were spaced 30 mm apart to achieve a similar intensity of competition to that in the tray experiments. Below the soil surface, pots were either undivided or divided into four equal sections using plastic strips, thus providing conditions in which below-ground competition was either allowed or prevented. Plants were grown either in monoculture (four plants of one genotype in the same pot) or in a two-way mixture containing two plants of each genotype with the same genotype at diagonally opposite corners of the pot. Plants were cultivated simultaneously in the same glasshouse from June to August 2011. Temperatures were quite variable (mean 20 °C, maximum 35 °C, standard deviation of mean temperature 3 °C). Measurements taken were the same as for plants in the four-way mixture experiment, with the addition of a rosette diameter measurement at 4 weeks old, which was not possible to do in the large, crowded tray experiments.

### Root growth assays

Seedling root growth assays were conducted to test if early root growth rates differed between genotypes. Thirty seedlings of all eight genotypes were grown on plates containing half-strength Murashige and Skoog (1/2 MS) medium in environmentally controlled cabinets (Snijders Economic Delux Dimmable containing Sylvania Britegro F36WT8/2084 bulbs). Cabinets were set to a 16 h photoperiod, 23/16 °C day/night temperature. Plants were grown as single plants. Total root length measurements were taken at 6 and 10 d growth using the image processing package Fiji (Schindelin *et al.*, 2012).

### Statistical analysis

#### Four-way mixture experiments

Seed mass and flowering time were analysed in linear mixed models to evaluate differences between monocultures and mixtures. Fixed factors included growing season, nutrient level (high/low), cultivation type (monoculture/mixture), genotype and their interactions. Seed mass was log-transformed to normalize the distribution of residuals and to make them approximately independent of fitted values. Non-significant interaction terms were removed from the model. Random effects were the block (tray) in which the plants were grown and the individual plants. Initially the model was run for the combined autumn and winter data set. The summer data set (including the additional heat stress) was analysed in a separate model. Finally, a model was run for all three data sets combined to assess the effect of the additional heat stress in the summer season on plant fitness in mixtures and monocultures. Details of the models are given in the Results. All unplanned two-way comparisons were tested by protected least significant difference (LSD).

#### Pair-wise interaction experiments

Initially a linear mixed model was run to test the strength of competition between genotypes within pots; fixed factors included competition (presence/absence of competitors) and the competitive group of the focal plant from which phenotypic measurements were taken. To evaluate differences between monocultures and mixtures in seed mass, rosette size and flowering time, a separate linear mixed model was then run on data from pots in which competitors were present. This included the main effect of each variable and the interactions between them. Fixed factors included genotype, competition type (above-ground only, or above- and below-ground competition), cultivation type (mixture/monoculture) and their interactions. Seed mass was log transformed, as above. A separate linear mixed model included the effect of competition type and competitive group (of the focal and the competing genotypes) on seed mass, rosette size and flowering time. Seed mass was square-root transformed to normalize the distribution of residuals and to make them approximately independent of fitted values. All non-significant interactions terms were removed from the model. Random effects in this model were the pot in which the plants were grown and the individual plants. All other factors were treated as fixed. All statistical analysis was conducted using Genstat v.12 (Payne, 2009).

## RESULTS

### Four-way mixture experiment

**Fig. 1.**
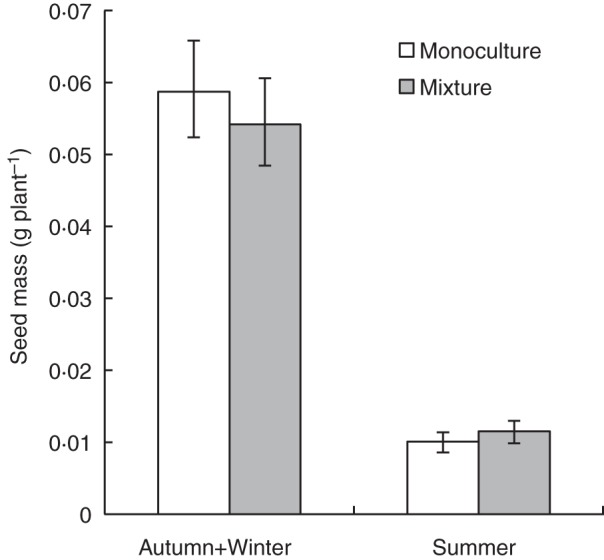
Mean seed yields of arabidopsis genotypes grown in mixtures and in monoculture in the four-way mixture experiments conducted during the autumn and winter seasons, and the summer season. *n* = 1880. Error bars = 95 % confidence interval.

**Fig. 2.**
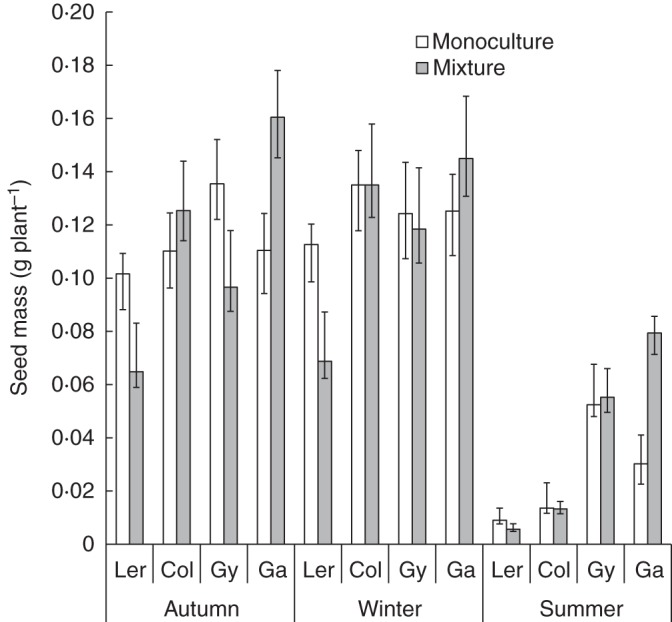
Mean seed production per plant of each genotype grown in monoculture or mixtures for each four-way mixture experiment. *n* = 1880. Error bars = 95 % confidence interval.

**Fig. 3.**
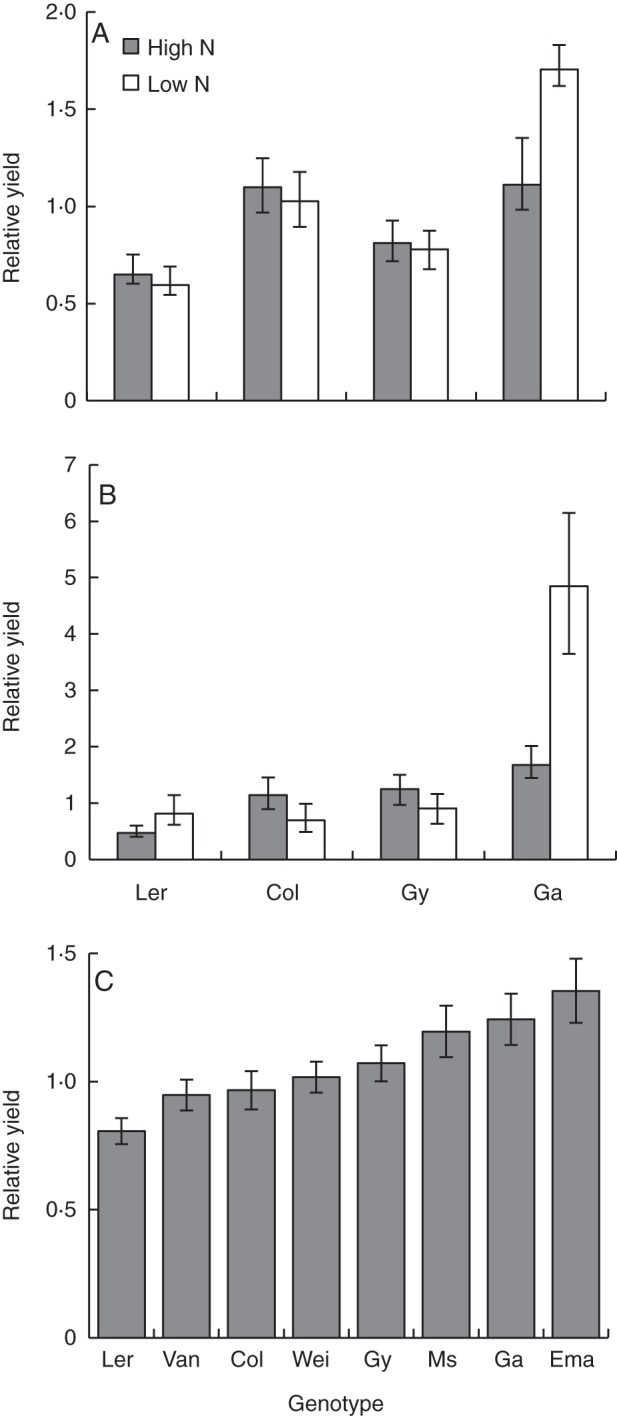
Relative yield (mixture yield/monoculture yield) for each arabidopsis genotype under high and low nutrient treatment in a four-way mixture experiment conducted during (A) the autumn and winter (*n* = 1260) and (B) the summer (*n* = 620). (C) Relative yields for eight genotypes in the pair-wise interaction experiment. Competitive groups of genotypes increase from left to right on the graph. *n* = 639. Error bars = 95 % confidence interval.

**Fig. 4.**
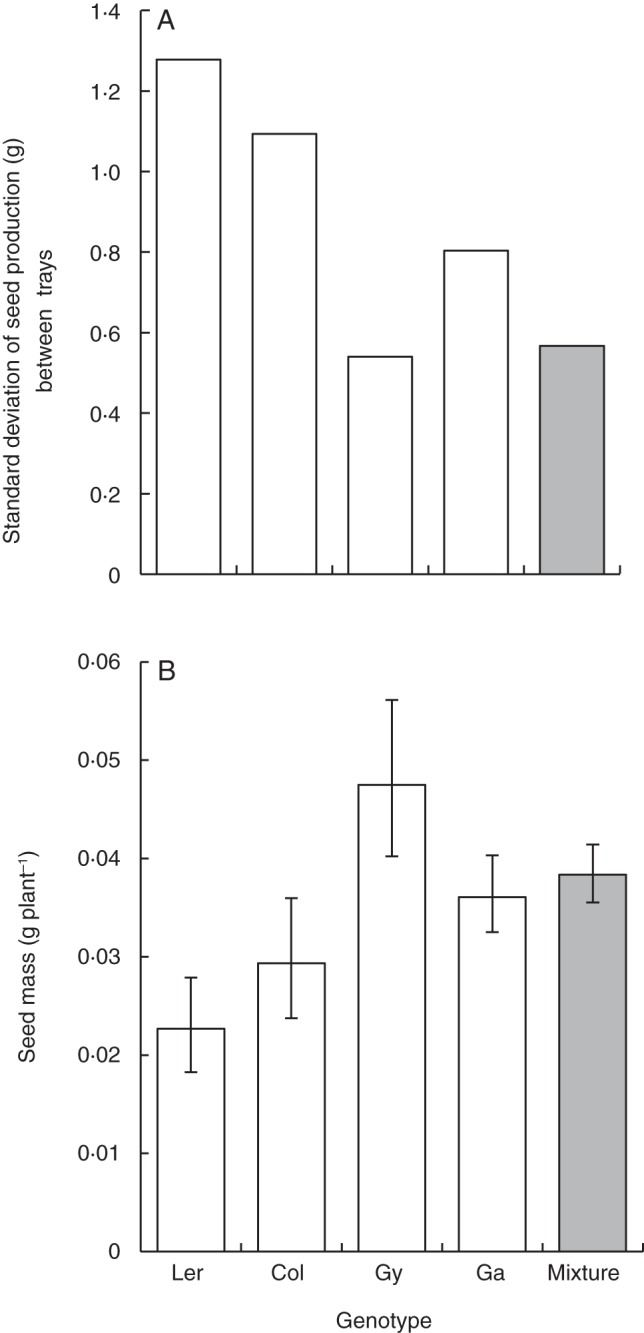
(A) Standard deviation of the mean seed mass produced per tray (block) in a four-way mixture experiment. (B) Mean plant yield in genotypic monoculture and the four-way mixture averaged over the entire experiment. *n* = 1880. Error bars = 95 % confidence interval.

Genotypic mixtures produced similar yields to those obtained in monocultures across all three experiments (Fig. 1; Supplementary Data Table S1a; *F*_1,39_ = 5·52, *P* = 0·02). Gy-0 achieved the highest yields in monoculture in two of the three experiments (Fig. 2). The genotype Ga-0 consistently produced more seed in mixtures (mean relative yield = 1·5), whereas Ler-1 consistently produced less seed in mixtures (mean relative yield = 0·6; Fig. 3A, B; Supplementary Data Table S1a; *F*_3,39_ = 25·29, *P* < 0·001). Despite differences in the yield of individual genotypes, the overall yield stability of mixtures (calculated by standard deviation) was approximately the same as that of the most stable genotype in monoculture (Fig. 4A).

The highly significant interaction between growing season and genotype reflects differential responses of the four genotypes to different glasshouse environmental conditions across the three seasons, in particular the summer experiment in which the plants were subjected to additional heat stress (Fig. 2; Supplementary Data Table S1a; *F*_6,39_ = 56·17, *P* < 0·001). To examine the effect of growing season, data from the summer experiment were separated from those of the autumn and winter experiments.

Genotype had the largest effect on seed production in the autumn and winter experiments (Fig. 2; Supplementary Data Table S1b; *F*_3,13_ = 67·7, *P* < 0·001), while the effect of growing season (autumn/winter) was comparatively small (*F*_1,13_ = 2·12, *P* = 0·02). The significant interaction between cultivation type (mixture/monoculture) and genotype in the autumn and winter experiments (Fig. 2; Supplementary Data Table S1b; *F*_3,13_ = 16·23, *P* < 0·001) reflects differential responses of the four arabidopsis genotypes to the two cultivation types in which they were grown. As expected, plants produced more seed under high nutrient conditions (Supplementary Data Fig. S2; Table S1b; *F*_1,13_ = 12·53, *P* < 0·001).

The additional heat stress substantially reduced growth of Col-0 and Ler-1 in the summer experiment. In the other two seasons, Ler-1 and Col-0 were 82 and 60 % taller, respectively (Supplementary Data Fig. S4). Genotype had the largest effect on seed production in summer (Fig. 2; Supplementary Data Table S1c; *F*_3,15_ = 156·92, *P* < 0·001). Despite the additional heat stress in summer, genotype performance was qualitatively similar across the entire experiment; in particular, Ga-0 consistently over-yielded in mixtures (Fig. 3A, B, relative yield >1). However, there were substantial quantitative differences between the summer experiment and the other two experiments. In the summer, plants produced much less seed (Fig. 1, 72 % overall reduction in seed production). There was a larger effect of cultivation method on seed production in summer (Supplementary Data Table S1c; *F*_4,15_ = 39·17, *P* < 0·001), largely because Ga-0 individuals receiving the low nutrient treatment produced significantly less seed in monoculture than they did in mixtures (Fig. 3A, B; *P <* 0·01 for difference from 1, LSD).

The number of days taken to flower differed between genotypes (Supplementary Data Fig. S3; Table S2; *F*_3,47_ = 876·13, *P* < 0·001) and between seasons (*F*_2,47_ = 284·45, *P* < 0·001), with some interaction between the two factors (*F*_6,47_ = 45·47, *P* < 0·001). There was an overall reduction in days taken to flower in the summer season (Supplementary Data Fig. S3). There was a small but significant interaction between genotype and cultivation method (Supplementary Data Table S2; *F*_3,47_ = 10·30 *P* < 0·001), attributable to slightly delayed flowering of Ga-0 in mixtures (Supplementary Data Fig. S3; *P* < 0·01, LSD). Gy-0 showed a delay in flowering when under low nutrient conditions in the summer which led to an unexpected increase in seed production (Supplementary Data Figs S2 and S3).

### Pair-wise interaction experiment

**Fig. 5.**
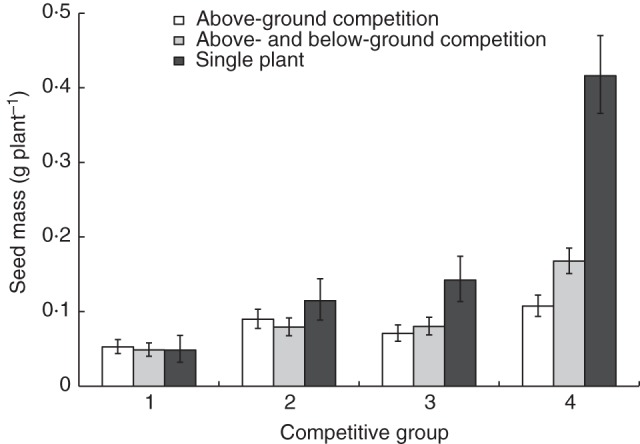
Mean seed production of focal arabidopsis plants from four competitive groups (1 = least competitive, 4 = most competitive) under three competition treatments (above-ground competition only, above- and below-ground competition, single plant) in a pair-wise interaction experiment. *n* = 639. Error bars = 95 % confidence interval.

**Fig. 6.**
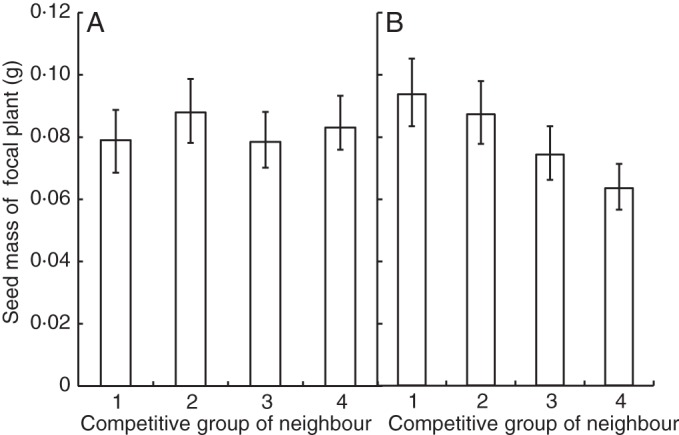
Mean seed production of focal arabidopsis plants grown with plants of four competitive groups (1 = least competitive, 4 = most competitive) in a pair-wise interaction experiment, (A**)** when competition was restricted to above-ground only, (B**)** when competition occurred both above- and below-ground. For competitive groups, see Table 1. *n* = 639. Error bars = 95 % confidence interval.

Competition was studied in the absence and presence of below-ground competition to test whether above-ground traits or below-ground traits had the greatest effect on competitive ability. Genotype had the greatest effect on seed production (Supplementary Data Table S3; *F*_7,24_ = 137·76, *P* < 0·001). There was a small overall effect of competition type (either above-ground competition only or both above- and below-ground competition) on seed production, largely due to an interaction between competition type and genotype (Supplementary Data Table S3; *F*_14,24_ = 7·91, *P* < 0·001). Mixtures achieved slightly greater yields than monocultures (Supplementary Data Fig. S5, Table S3; *F*_1,24_ = 9·87, *P* < 0·001). Mixture performance of genotypes increased with competitive group (Fig. 3C). The factor affecting seed production most strongly was the phenotype of the focal plant, as large rosette size (*x*) was consistently associated with increased seed production (*y* = 301·41*x* + 30·72, *R*^2^ = 0·55). The competitive group of both the focal plant (Supplementary Data Table S4; *F*_3,13_ = 143·6, *P* < 0·001) and the competing plant (Supplementary Data Table S4; *F*_3,13_ = 6·16, *P* < 0·001) significantly affected seed production. More competitive groups showed a larger reduction in seed production in the presence of competition (Fig. 5; Supplementary Data Table S5; *F*_3,7_ = 16·1, *P* = 0·001), indicating that these highly competitive genotypes have the greatest yield potential, and the ability to utilize limited resources allows them to over-yield in mixture, but they may not perform so well in monoculture. Yield of the focal plant decreased when the competitive ability of the neighbour increased, but only when competition was unrestricted (Fig. 6; Supplementary Data Table S4; *F*_3,13_ = 3·77, *P* = 0·01).

### Root growth assays

Seedling root growth assays showed no significant effect of genotype on initial root growth rates (Supplementary Data Fig. S6) although the conditions in which root growth was measured were inevitably not the same as those used in the glasshouse, where the plants were grown in soil.

## DISCUSSION

We investigated the suitability of *Arabidopsis thaliana* as an ecological model for studying intraspecific competition between plants at different levels of genetic and phenotypic diversity. In this study, arabidopsis genotypic diversity enhanced ecological resistance of the population to nutrient stress and the combination of nutrient and heat stress shown by an increase in yield and yield stability compared with the average monoculture. Mixtures produced yields that were as stable and almost as high as the best performing monoculture (Gy-0) over the entire experiment (Fig. 4A, B), supporting the hypothesis that biodiversity increases ecological stability (Yachi and Loreau, 1999; Hooper *et al.*, 2005; Tilman *et al.*, 2006). Yield stability was achieved through compensation in which the fittest, most plastic genotype with high yield potential (e.g. Ga-0) over-yielded in genetic mixtures, compensating for the lower yield of less fit genotypes (e.g. Ler-1) (Figs 3A, B and 4; Supplementary Data Table S1). This effect was greatest in the summer experiment when plants were under the highest levels of abiotic stress. Compensation was seen throughout the study despite genotypic variation in responses to environmental conditions. There was no transgressive over-yielding, which would have been an indication of complementary resource usage, and plants always performed better in the absence of others, indicating that facilitation did not occur (Fig. 4B) (Callaway, 2007; Brooker, 2008). As complementation was not detectable in this experiment, we conclude that compensation was responsible for the increased ecological resistance of arabidopsis mixtures to nutrient stress and also the combination of nutrient and heat stress.

The role of root competition in plant genetic mixtures is intriguing and appears to have been important in this experiment. Although competition for space above-ground is obvious, the results of the pair-wise interaction experiments indicate that, in fact, competition between arabidopsis plants depends more on below-ground interactions. The most competitive genotypes decreased the yield of focal plants only when below-ground competition was permitted, indicating that below-ground competition may be more important than above-ground competition in arabidopsis when securing resources for seed production (Fig. 6; Supplementary Data Table S3). The growth habit of the arabidopsis rosette prevented the partitioning of the aerial space in a similar way to that done for the soil space, a common method for separating above- and below-ground competition (Semere and Froud-Williams, 2001; Cahill, 2002). Restricting competition with partitions can also create artificial effects including alteration of the root system architecture (McPhee and Aarssen, 2001). No significant interaction was identified between competitive ability and seedling root growth, indicating that some property of adult plant roots allows certain genotypes to outcompete others for below-ground resources (Supplementary Data Fig. S6). Below-ground competition for nutrients, water and space often affects plant growth more than above-ground competition, yet it remains overlooked in many studies of competition between plants (Casper and Jackson, 1997). This study implies that it is crucial to understand below-ground interactions between adult plants in order to predict accurately the outcome of competition between cultivars and design sustainable cropping systems.

Nevertheless, the competitive ability of genotypes was predictable from the above-ground phenotype. The most competitive genotypes had larger rosettes, took longer to flower, were more plastic in their flowering time and produced more seed, confirming predictions from the four-way mixture experiment. These results suggest that competitive ability can be predicted in crops prior to competition experiments. Such data can be used to estimate the mixing ability of genotypes and increase the efficiency of mixture selection (Knott and Mundt, 1990). Certain genotypes may contribute more yield in mixtures than in monoculture; for example, in the four-way mixture experiment, Gy-0 monocultures produced more seed than Ga-0 monocultures yet Ga-0 was the highest yielding genotype in mixtures (Fig. 4A). This implies that high levels of intragenotypic competition decreased the yield of individual Ga-0 plants in monoculture, indicating that while Ga-0 is a strong competitor with other genotypes, it is not well adapted to intragenotypic competition. This effect was amplified under low nutrient conditions where Ga-0 showed a significant reduction in yield when grown in monoculture compared with the mixture. The phenotypic plasticity of Ga-0 (e.g. a delay in flowering time in mixtures) allowed the genotype to respond to different growing conditions in a way that the more static Gy-0 did not. Under less predictable environmental conditions (seen in the summer experiment), phenotypic plasticity and high yield potential enable genotypes such as Ga-0 to compensate for less fit genotypes, thereby increasing yield stability through enhanced resistance.

The pair-wise interaction experiments suggested little advantage of being in the lowest competitive groups (group 1 and 2), but their reduced time taken to flower may be advantageous in very unpredictable environments in which setting seed quickly provides escape from potentially fatal environmental conditions. We speculate that if an additional drought stress was included in the summer experiment, then Ga-0 and Gy-0 individuals would have died before setting seed. These experiments showed the seed production of group 4 genotypes to be most restricted by the presence of competition, a trait that increases the potential for compensation in mixtures via competitive release (Fig. 5; Supplementary Data Table S5). This finding highlights the importance of mixture selection because successful mixtures must contain components that are not only good performers but also good neighbours (Mundt *et al.*, 1995).

To date, the majority of studies of genetic mixtures in agriculture have been conducted under field conditions and have focused on the ability of mixtures to control disease (Finckh *et al.*, 2000; Zhu *et al.*, 2000; Mundt, 2002). Varietal mixture studies often report general trends in yield and disease severity for the population (Mundt, 2002; Philips *et al.*, 2005; Newton and Guy, 2009), but few studies have focused on the plant–plant interactions taking place within mixtures (Allard and Adams, 1969; Finckh and Mundt, 1992). Empirical studies that attempt to separate the effects of abiotic and biotic stress on mixtures are impractical because of the unique environmental conditions of the field (Finckh *et al.*, 2000). Arabidopsis provides a model system in which individual stresses can be applied separately and in combination, and in which genotype by environment interactions can be closely studied under environmentally controlled conditions in an efficient and repeatable way. In this study, arabidopsis provided insight into the mechanisms of plant competition within genetic mixtures and demonstrated its potential in ecological research.

The arabidopsis model system can be used to study the ecological genetics of crops and their responses to pathogens, pests and weeds, all of which will become increasingly important as the chemicals used to control them become more heavily regulated. Studies investigating competitive ability of varieties will become increasingly relevant to productivity if selection for increased competitive ability against weeds has to be traded off against less competition between plants in order to maintain yield (Jordan, 1993; Lemerle *et al.*, 2006; Song, 2010). Cropping systems will need to be less reliant on chemical input, less expensive to manage and show greater adaptability to the changing environment if future food security is to be achieved (FAO, WFP, IFAD, 2012; Hillocks, 2012). Genetically diverse crops, able to adapt to a wider range of environments, will contribute to stable, high productivity by buffering against diverse and sometimes unpredictable stresses.

## SUPPLEMENTARY DATA

Supplementary data are available online at www.aob.oxford-journals.org and consist of the following. Table S1: the effect of arabidopsis genotypic diversity and different growing conditions on seed productivity in the four-way mixture experiments. Table S2: the effect of arabidopsis genotypic diversity and different growing conditions on days to flowering in a four-way mixture experiment. Table S3: the effect of arabidopsis genotypic diversity and different growing conditions on seed productivity in a pair-wise interaction experiment. Table S4: the effect of competitive group of the focal and competing plant, competition type (above-ground only/above- and below-ground) on seed productivity of arabidopsis plants in the pair-wise interaction experiments. Table S5: the effect of competition (presence/absence of competitors) and competitive group of the focal plant on seed productivity of arabidopsis plants in a pair-wise interaction experiment. Figure S1: planting design for four-way mixtures of arabidopsis genotypes. Figure S2: mean seed production per plants of each genotype under high or low nutrient treatment for each experiment. Figure S3: mean days taken to flower for four arabidopsis genotypes grown in monoculture and mixture in a four-way mixture experiment. Figure S4: mean plant height for each genotype grown in each experiment. Figure S5: mean seed production in mixtures and monocultures in the pair-wise interaction experiments. Figure S6: seedling root growth after 10 d growth on 1/2 MS plates.

Supplementary Data
