# Supplementary material for: Stabilization of yield in plant genotype mixtures through compensation rather than complementation
Source: Ann Bot. 2013 Sep 18;112(7):1439–47. doi: 10.1093/aob/mct209 (PMC3806538; doi:10.1093/aob/mct209)
Supplement: Supplementary Data [file supp_112_7_1439_v2_index.html]

Stabilization of yield in plant genotype mixtures through compensation rather than complementation — Supplementary Data 

# Stabilization of yield in plant genotype mixtures through compensation rather than complementation

## Supplementary Data

Supplementary Data

**Files in this Data Supplement:**

- Supplementary Figures - pdf file
- Supplementary Tables - pdf file
