# Supplementary material for: Stabilization of yield in plant genotype mixtures through compensation rather than complementation
Source: Ann Bot. 2013 Sep 18;112(7):1439–47. doi: 10.1093/aob/mct209 (PMC3806538; doi:10.1093/aob/mct209)

Fig. S1. Planting design for four-way mixtures of *Arabidopsis* genotypes.

A	B	C	D	A	B	C	D	A	B	C	D
C	D	A	B	C	D	A	B	C	D	A	B
B	A	C	D	B	A	C	D	B	A	C	D
C	D	B	A	C	D	B	A	C	D	B	A
B	A	D	C	B	A	D	C	B	A	D	C
D	C	B	A	D	C	B	A	D	C	B	A
A	B	D	C	A	B	D	C	A	B	D	C
D	C	A	B	D	C	A	B	D	C	A	B
C	A	B	D	C	A	B	D	C	A	B	D
B	D	C	A	B	D	C	A	B	D	C	A
A	B	D	C	A	B	D	C	A	B	D	C
D	C	A	B	D	C	A	B	D	C	A	B
A	B	D	C	A	B	D	C	A	B	D	C
C	A	B	D	C	A	B	D	C	A	B	D
B	D	C	A	B	D	C	A	B	D	C	A

Fig. S2. Mean seed production per plants of each genotype under high or low nutrient treatment for each experiment.

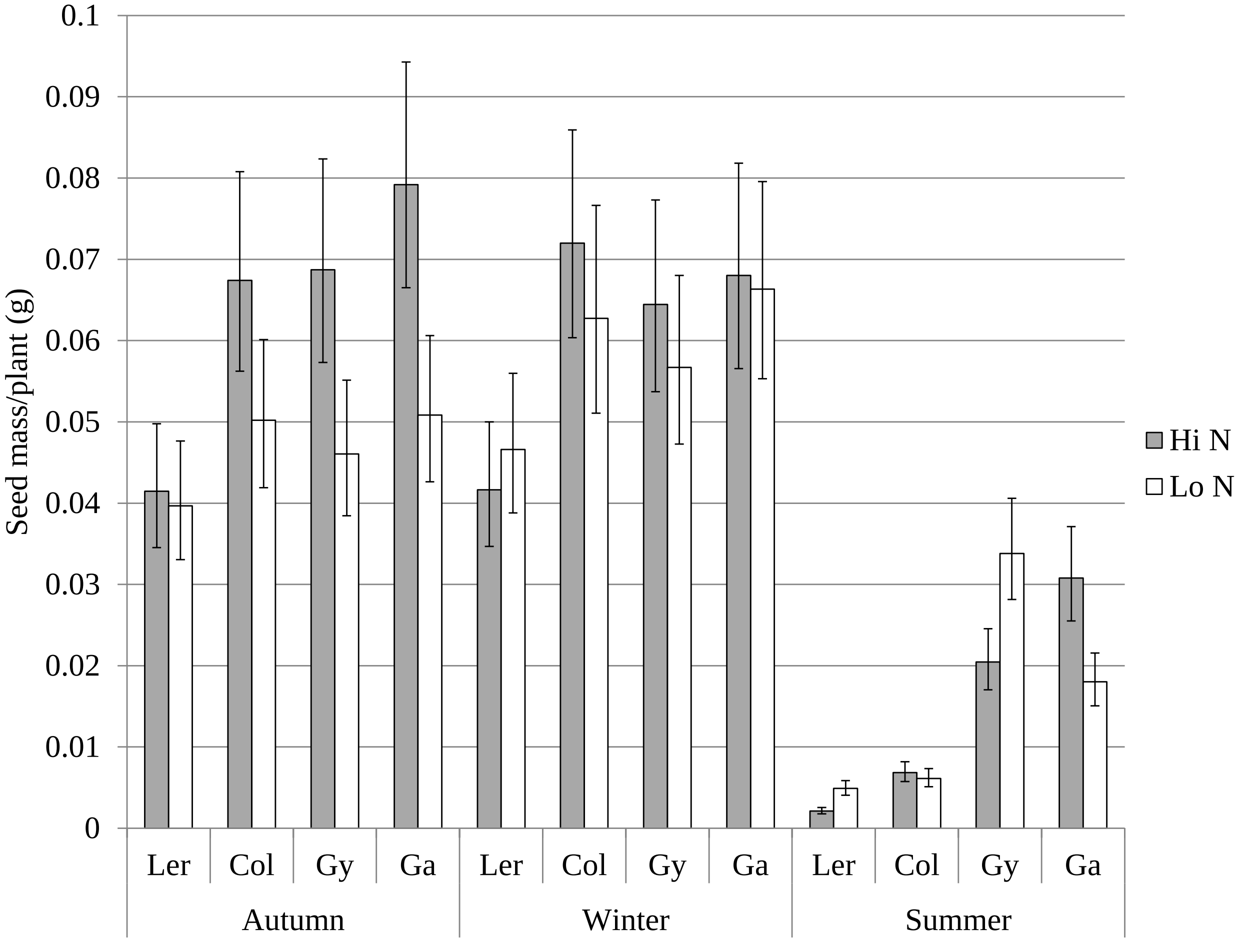

Fig. S3. Mean days taken to flower for four *Arabidopsis* genotypes grown in monoculture and mixture in a four-way mixture experiment.

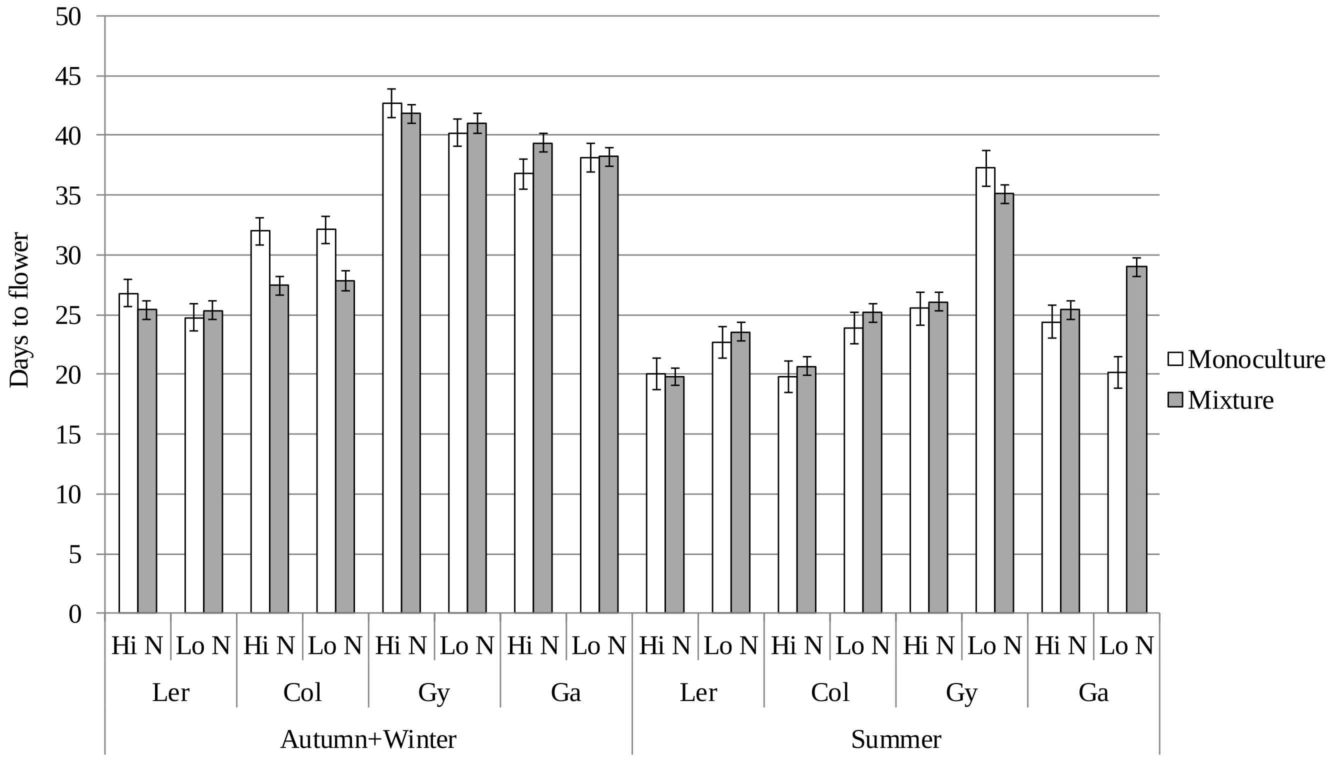

Fig. S4. Mean plant height for each genotype grown in each experiment.

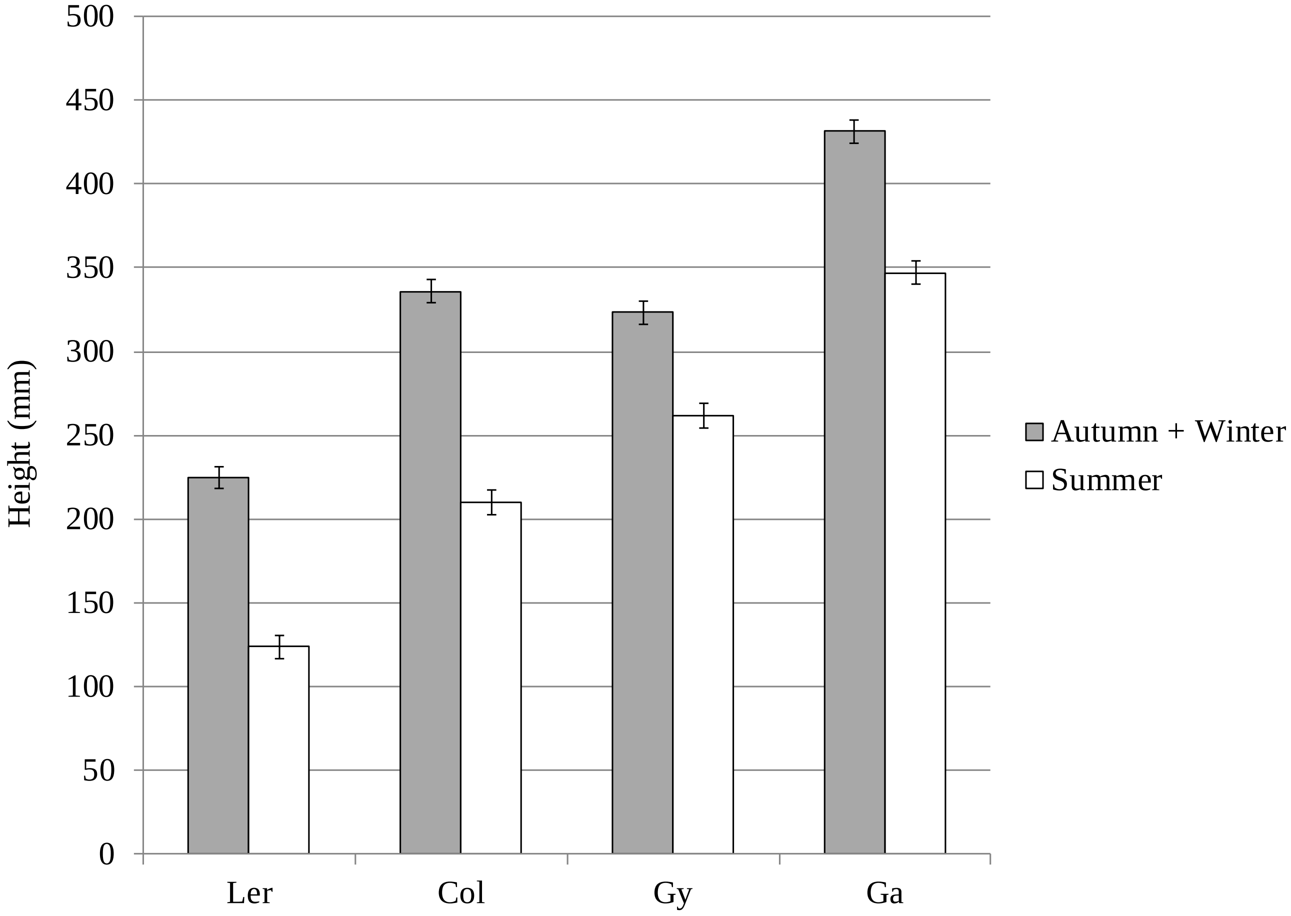

Fig. S5. Mean seed production in mixtures and monocultures in the pair-wise interaction experiments.

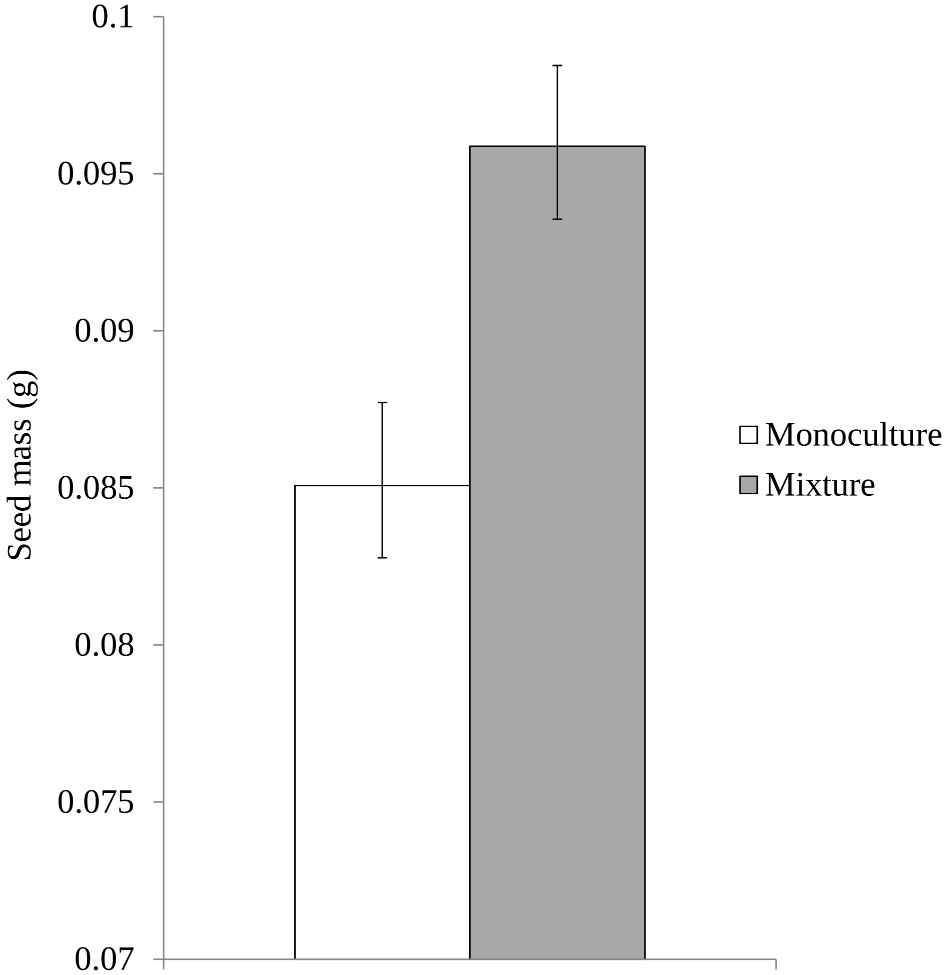

Fig. S6: Seedling root growth after ten days growth on ½ MS plates.

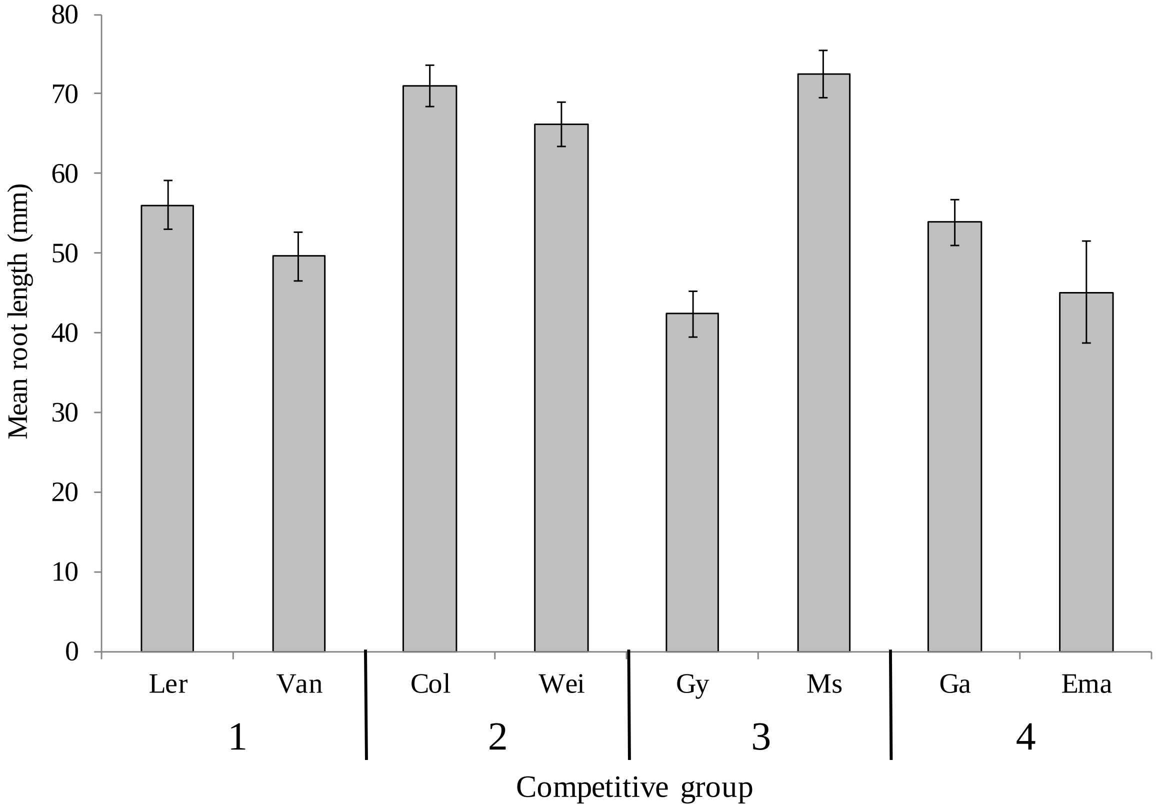

Supplement: Supplementary Data [file supp_mct209_mct209supp_figs.pdf]
