# Supplementary material for: Stabilization of yield in plant genotype mixtures through compensation rather than complementation
Source: Ann Bot. 2013 Sep 18;112(7):1439–47. doi: 10.1093/aob/mct209 (PMC3806538; doi:10.1093/aob/mct209)
Supplement: Supplementary Data [file supp_mct209_mct209supp_tables.pdf]

**Table S1.** The effect of *Arabidopsis* genotypic diversity and different growing conditions on seed productivity in the four-way mixture experiments. In each case, a linear mixed model was used to analyse each factor and the interactions between them. Fixed effects included growing season (autumn, winter, summer), nutrient level (high/low) and cultivation (monoculture/mixture) and genotype. Non-significant interaction terms were removed from each model. F: variance ratio for use in F-test; n.d.f.: numerator degrees of freedom; d.d.f.: denominator degrees of freedom.

a) Analysis of combined data from all three experiments. N=1880.

| Fixed effect | F | n.d.f. | d.d.f. | P |
|---|---|---|---|---|
| season | 305.66 | 2 | 63.5 | <0.001 |
| nutrient level | 0.92 | 1 | 60.3 | 0.3 |
| cultivation | 5.52 | 1 | 61.7 | 0.02 |
| genotype | 153.45 | 3 | 940.5 | <0.001 |
| season x nutrient | 11.99 | 2 | 65.3 | <0.001 |
| season x cultivation | 13.39 | 2 | 66.9 | <0.001 |
| genotype x season | 56.17 | 6 | 888.6 | <0.001 |
| genotype x nutrient level | 5.26 | 3 | 921.1 | 0.001 |
| genotype x cultivation | 25.29 | 3 | 135.4 | <0.001 |
| genotype x season x nutrient level | 4.48 | 6 | 834.9 | <0.001 |
| genotype x season x cultivation | 2.48 | 6 | 157.4 | 0.03 |
| genotype x nutrient level x cultivation | 3.68 | 4 | 108.4 | 0.007 |

b) Analysis of data from the autumn and winter seasons of the four-way mixture experiment. N=1260.

| Fixed term | F | n.d.f. | d.d.f. | P |
|---|---|---|---|---|
| season | 2.12 | 1 | 47.5 | 0.02 |
| nutrient level | 12.53 | 1 | 46.5 | <0.001 |
| cultivation | 2.39 | 1 | 47.3 | 0.1 |
| genotype | 67.7 | 3 | 750.4 | <0.001 |
| season x nutrient level | 5.43 | 1 | 47.1 | 0.02 |
| genotype x nutrient level | 3.14 | 3 | 751.4 | 0.03 |
| genotype x cultivation | 16.23 | 3 | 105.4 | <0.001 |

c) Analysis of data from the summer season of the four-way mixture experiment. N= 620.

| Fixed term | F | n.d.f. | d.d.f. | P |
|---|---|---|---|---|
| nutrient level | 10.66 | 1 | 21.0 | 0.004 |
| cultivation | 39.17 | 1 | 23.0 | <0.001 |
| genotype | 156.92 | 3 | 184.8 | <0.001 |
| genotype x nutrient level | 7.43 | 3 | 159.1 | <0.001 |
| genotype x cultivation | 11.82 | 3 | 62.0 | <0.001 |
| genotype x nutrient level x cultivation | 3.22 | 4 | 49.9 | 0.02 |

**Table S2.** The effect of *Arabidopsis* genotypic diversity and different growing conditions on days to flowering in all three growing seasons of a four-way mixture experiment. A linear mixed model analysed each factor individually and the interactions between them. Fixed factors include experimental season, nutrient level (high/low) and cultivation (monoculture/mixture) and genotype. N=1880. F: variance ratio for use in F-test; n.d.f.: numerator degrees of freedom; d.d.f.: denominator degrees of freedom.

| Fixed effect | F | n.d.f. | d.d.f. | P |
|---|---|---|---|---|
| season | 284.45 | 2 | 55.5 | <0.001 |
| nutrient level | 9.60 | 1 | 52.9 | 0.003 |
| cultivation | 0.01 | 1 | 56.9 | 0.9 |
| genotype | 876.13 | 3 | 1125.3 | <0.001 |
| season x nutrient level | 20.58 | 2 | 53.8 | <0.001 |
| season x genotype | 45.47 | 6 | 1204.2 | <0.001 |
| nutrient x genotype | 3.80 | 3 | 1124.0 | 0.01 |
| cultivation x genotype | 10.30 | 3 | 124.6 | <0.001 |
| season x nutrient level x genotype | 7.46 | 6 | 1097.0 | <0.001 |
| season x cultivation x genotype | 13.17 | 8 | 105.0 | <0.001 |
| season x nutrient level cultivation x genotype | 2.01 | 12 | 102.5 | 0.03 |

**Table S3.** The effect of *Arabidopsis* genotypic diversity and different growing conditions on seed productivity in a pair-wise interaction experiment. A linear mixed model was used to analyse each factor and all interactions between them. Fixed effects included genotype, competition type (above ground only/above and below ground) and cultivation (mixture/monoculture). Non-significant terms were eliminated from the model. *F* and *P* values refer to ANOVA tests of each factor separately and the interactions between them. N=639.

| Fixed term | F | n.d.f. | d.d.f. | P |
|---|---|---|---|---|
| genotype | 137.76 | 7 | 275.3 | <0.001 |
| competition type | 23.47 | 2 | 173.2 | <0.001 |
| cultivation | 9.87 | 1 | 287.2 | <0.001 |
| genotype x competition type | 7.91 | 14 | 176.7 | <0.001 |

**Table S4.** The effect of competitive group of the focal and competing plant, competition type (above ground only/above and below ground) on seed productivity of *Arabidopsis* plants in the pair-wise interaction experiments. A linear mixed model was used to analyse each factor and the interactions between them. Fixed effects included growing season, competition type (above/below-ground), competitive group of focal plant competitive group of competing plant. Non-significant interaction terms were removed from each model. *F* and *P* values refer to ANOVA tests of each factor separately and the interactions between them. N=639.

| Fixed term | F | n.d.f. | d.d.f. | P |
|---|---|---|---|---|
| competition type | 4.58 | 1 | 295.5 | 0.03 |
| competitive group of focal plant | 143.6 | 3 | 295.6 | <0.001 |
| competitive group of competing plant | 6.16 | 3 | 295.5 | <0.001 |
| competition type x competitive group of focal plant | 19.92 | 3 | 295.7 | <0.001 |
| competition type x competitive group of competing plant | 3.77 | 3 | 295.5 | 0.01 |

**Table S5.** The effect of competition (presence/absence of competitors) and competitive group of the focal plant on seed productivity of *Arabidopsis* plants in a pair-wise interaction experiment. A linear mixed model was used to analyse each factor and all interactions between them. Fixed effects included growing season, competition type (above/below-ground), competitive group of focal plant competitive group of competing plant. Non-significant interaction terms were removed from each model. *F* and *P* values refer to ANOVA tests of each factor separately and the interactions between them. N=639.

| Fixed term | F | n.d.f. | d.d.f. | P |
|---|---|---|---|---|
| competition (presence/absence) | 18.39 | 1 | 212.0 | <0.001 |
| competitive group of focal plant | 347.7 | 3 | 308.5 | <0.001 |
| competition (presence/absence) x competitive group of focal plant | 16.1 | 3 | 212.1 | 0.001 |
